# Long-term outcomes of vascularized fibular grafting for infected non-union of the lower limb: a competing-risk analysis

**DOI:** 10.5194/jbji-11-579-2026

**Published:** 2026-09-15

**Authors:** Anne Le Hir, Piseth Seng, David Delarbre, Romain Ambrosino, Najib Kachouh, Régis Legré, Andreas Stein

**Affiliations:** 1 Department of Infectious Diseases, Tropical Medicine and Chronic Infections (MIT-IC), IHU Méditerranée Infection, AP-HM, La Timone University Hospital, 19-21 Boulevard Jean Moulin, 13005 Marseille, France; 2 Aix-Marseille Université, Microbes Evolution Phylogeny and Infection (MEPHI), IHU Mediterranean Infection, 19-21 Boulevard Jean Moulin, 13005 Marseille, France; 3 Department of Internal Medicine, University Military Hospital Sainte-Anne, 2, Boulevard Sainte Anne, BP 600, 83000 Toulon, France; 4 Aix-Marseille Université, APHM, CNRS, ISM, CHU Timone, Service de chirurgie rachidienne, 264 rue Saint-Pierre, 13005 Marseille, France; 5 Department of Hand Surgery and Plastic and Reconstructive Surgery of the Limbs, La Timone University Hospital, Assistance Publique Hôpitaux de Marseille, 13005 Marseille, France

## Abstract

**Background**: Infected non-union of the lower limb is among the most challenging conditions in orthopedic surgery. Vascularized fibular grafting (VFG) is a limb-salvage option for large infected bone defects, but outcomes accounting for competing events remain poorly documented. **Methods**: We conducted a retrospective single-center study of adults treated consecutively with VFG for infected femoral or tibial non-union between 2014 and 2023. Infected non-union combined institutional non-union criteria with fracture-related infection as per international consensus definitions. Treatment success was defined as consolidation with infection remission. Amputation and death were competing events; cumulative incidence of consolidation was estimated using competing-risk methods. **Results**: A total of 22 patients were included (median age of 45 years). Consolidation was achieved in 17 patients (77 %), infection remission was achieved in 16 (73 %), and treatment success was achieved in 16 (73 %): 9 (41 %) after VFG alone and 7 (32 %) after secondary bone grafting. Six patients (27 %) had unfavorable outcomes: four amputations, one persistent non-union, and one death. The median time to consolidation was 14 months after VFG alone and 38 months after secondary grafting. Cumulative incidence of consolidation reached approximately 18 % at 12 months, 41 % at 24 months, 59 % at 36 months, and 76 % by end of follow-up. All seven patients undergoing secondary grafting for delayed consolidation ultimately achieved union; no formal comparative analysis was performed given the small, time-dependent sample. **Conclusion**: VFG is a valuable limb-salvage strategy for selected patients with infected non-union. Consolidation may occur late, underscoring the need for prolonged follow-up and competing-risk methodology. Findings should be interpreted given the retrospective design, small sample, and heterogeneous follow-up.

## 1 Introduction

Infected non-union represents one of the most severe complications of fracture management, combining failure of bone healing with persistent infection. In contemporary practice, infected non-union is generally considered to be a manifestation of fracture-related infection (FRI) associated with the absence of bone consolidation and is diagnosed according to the international FRI consensus definition, which incorporates clinical, microbiological, histopathological, and radiological criteria (Metsemakers et al., 2018; McNally et al., 2020). Infected non-union is associated with poor clinical outcomes and remains among the most complex forms of FRI.

Several risk factors contribute to the development of infected non-union. These include patient-related factors such as smoking, diabetes mellitus, obesity, and peripheral arterial disease, as well as trauma-related factors, particularly open fractures, which increase the risk of contamination and subsequent infection (Cicero-Álvarez et al., 2016; Jensen et al., 2022).

Fracture non-union results in chronic pain, functional impairment, and prolonged disability. When infection is present, spontaneous bone healing becomes unlikely because bacterial biofilm formation on osteosynthesis material impairs both host immune responses and antibiotic efficacy. Consequently, management often requires multiple surgical procedures combined with prolonged systemic antibiotic therapy.

In severe cases, amputation may be considered to be a definitive treatment option. However, limb loss is associated with major functional and psychological consequences despite significant improvements in prosthetic technology (Sahu et al., 2017). In infected non-union of long bones, extensive debridement and segmental bone resection may be required to eradicate infection, resulting in large bone defects that necessitate complex reconstructive procedures.

Three major reconstructive strategies are commonly used for the management of segmental bone defects in infected non-union: the Masquelet induced-membrane technique, distraction osteogenesis with bone transport, and vascularized bone grafts (Sidiropoulos et al., 2023). The Masquelet technique relies on a two-stage procedure involving the formation of an induced membrane followed by bone grafting, with reported success rates of around 69 % in infected non-union (Giovanoulis et al., 2023). Bone transport techniques, originally popularized using circular external fixation, can also be performed using monolateral fixation systems or motorized intramedullary nails and remain a cornerstone of reconstruction in FRI-associated bone defects (Metsemakers et al., 2026; Sidiropoulos et al., 2023). Both approaches rely primarily on the biological regenerative capacity of the recipient site and may be challenged by extensive scarring, compromised vascularity, or repeated surgical failures.

Vascularized fibular grafting (VFG), initially described in the 1970s, provides living bone with its own blood supply and has been widely used for the reconstruction of large bone defects exceeding 6 cm (Pederson and Person, 2007; Taylor et al., 1975; Xenakis et al., 1994). By restoring intrinsic vascularization, VFG improves local immune defense and antibiotic penetration at the infected site, which may be particularly advantageous in cases of infected non-union.

In our institution, VFG is generally considered to be a salvage procedure followed after the failure of conventional reconstructive techniques and before the consideration of major amputation. However, most studies evaluating outcomes rely on Kaplan–Meier survival analysis, which may overestimate consolidation rates because competing events such as amputation or death are treated as censored observations.

The aim of this study was therefore to evaluate the outcomes of VFG for infected non-union of the lower limb using a competing-risk framework and to explore the association between secondary bone grafting and bone consolidation.

## 2 Methods

### 2.1 Study design and setting

This retrospective, monocentric observational study was conducted in a tertiary referral center specializing in complex bone and joint infections between 1 January 2014 and 31 December 2023. The study aimed to evaluate the outcomes of vascularized fibular grafting (VFG) used as a salvage procedure for infected non-union of the lower limb.

### 2.2 Definition of infected non-union

Infected non-union was defined as the combination of a confirmed fracture-related infection (FRI) according to the international FRI Consensus Definition and established non-union (Govaert et al., 2020; Metsemakers et al., 2018). Non-union was defined according to our institutional criteria as failure of bone healing for at least 6 months, with no radiographic progression toward union during the preceding 3 months. At least one confirmatory criterion of FRI was required, including a sinus tract communicating with the fracture site, purulent drainage, phenotypically identical microorganisms isolated from at least two deep tissue cultures, or histopathological evidence of infection. At surgery, at least five deep tissue specimens were routinely obtained for microbiological analysis. Culture incubation was performed according to institutional protocols for a minimum of 10 d. 

### 2.3 Inclusion and exclusion criteria

Patients were eligible for inclusion if they met the following criteria: (1) vascularized fibular graft performed for infected non-union, (2) involvement of the lower limb (femur or tibia), (3) documented fracture-related infection according to the FRI consensus definition before VFG, and (4) minimum follow-up of 6 months after VFG. Although the minimum required follow-up was 6 months, no patient was censored between 6 and 12 months without experiencing consolidation, amputation, or death.

Patients were excluded if they were younger than 18 years or if VFG was performed for aseptic bone defects or primary oncologic reconstruction without infection. Patients who underwent oncologic reconstruction and subsequently developed a documented infection requiring VFG were eligible for inclusion.

### 2.4 Surgical procedure

**Figure 1:**
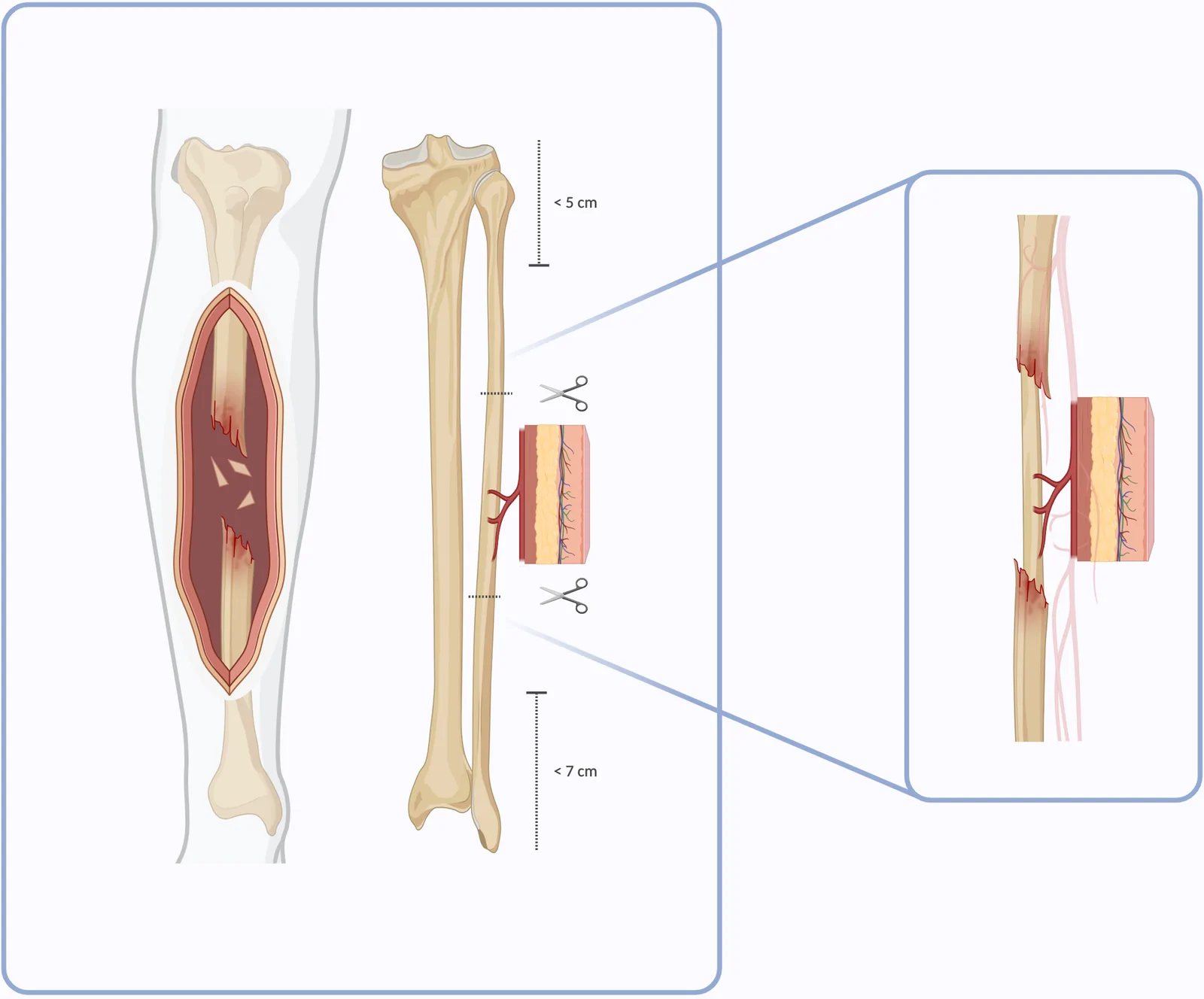
Schematic illustration of the surgical technique for vascularized fibular autograft reconstruction, including radical debridement, segmental bone resection, vascularized fibular graft harvest, microsurgical anastomosis, and bone fixation (created with https://BioRender.com, last access: 13 May 2025).

**Figure 2:**
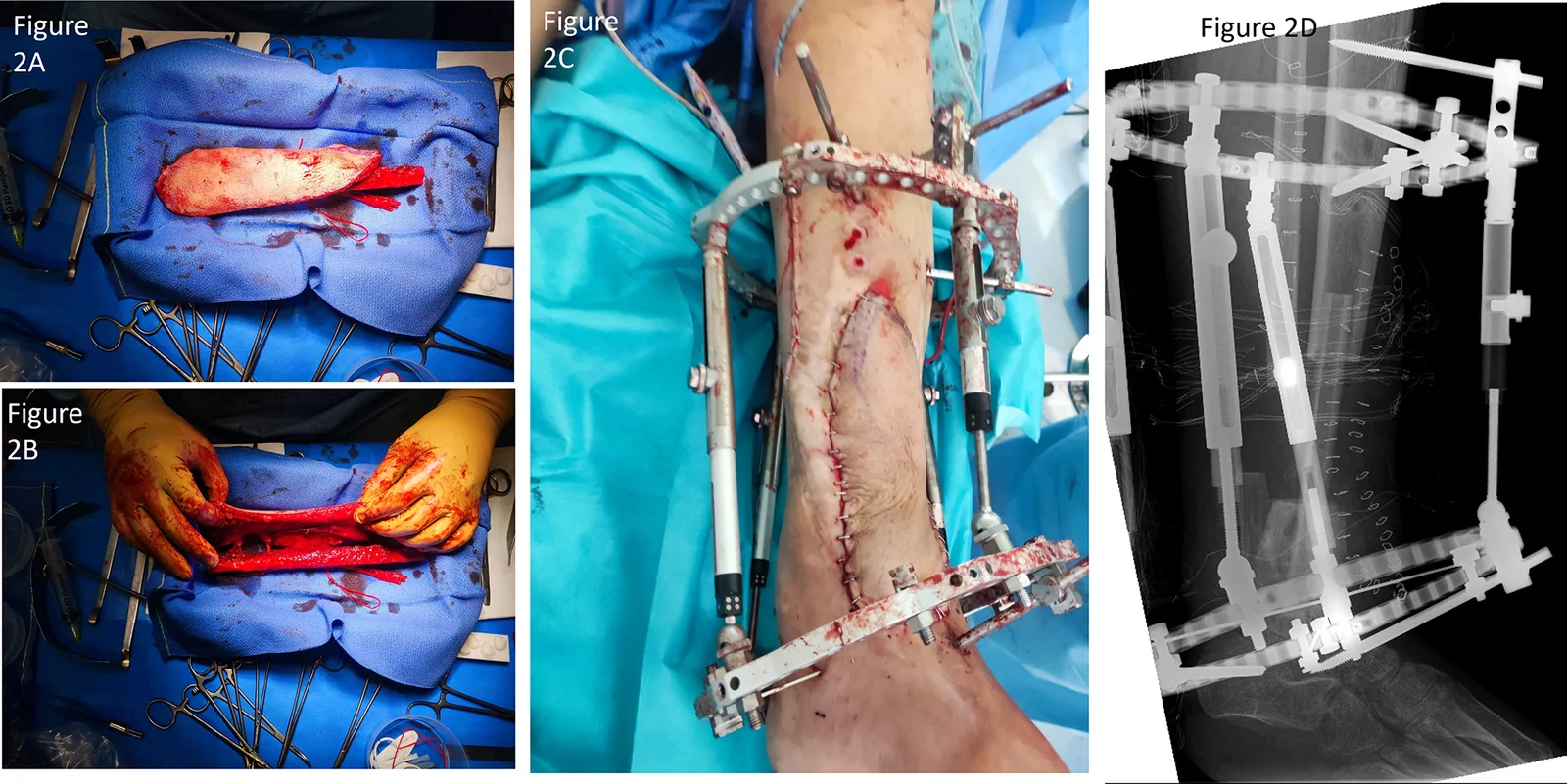
Representative case of vascularized fibular grafting (VFG) for infected non-union of the lower limb. **(A)** Harvest of a vascularized fibular osteocutaneous graft with skin paddle from the contralateral leg. **(B)** Isolated vascularized fibular graft after harvest. **(C)** Intraoperative view following implantation of the vascularized fibular graft at the recipient site. **(D)** Postoperative radiograph demonstrating graft positioning and fixation after VFG reconstruction.

**Figure 3:**
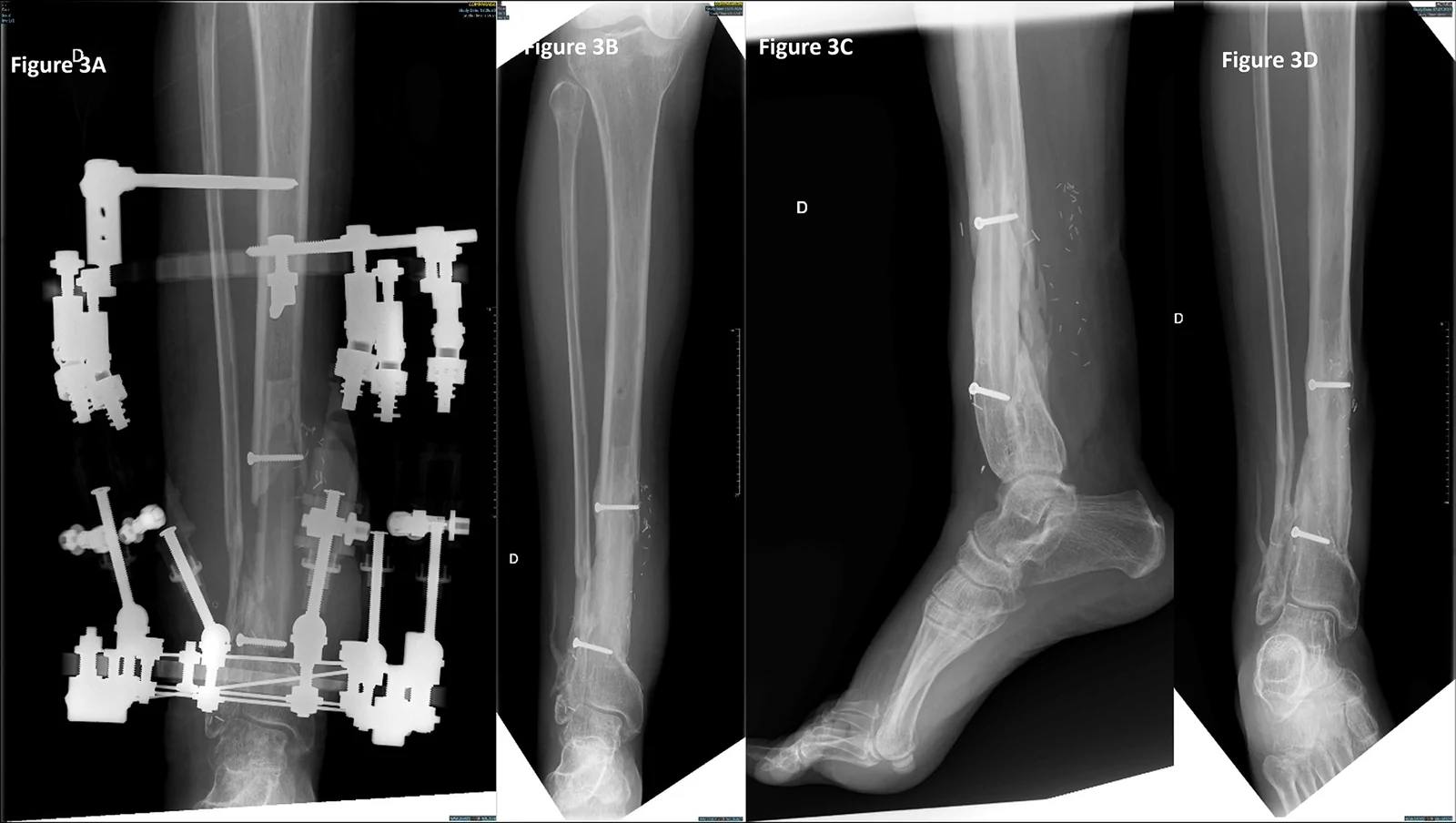
Serial radiographic evolution following vascularized fibular grafting (VFG) for infected non-union. **(A)** Immediate postoperative radiograph after VFG reconstruction. **(B)** Radiograph obtained 13 months after surgery showing progressive graft incorporation. **(C, D)** Radiographs obtained 21 months after VFG demonstrating advanced graft hypertrophy, cortical integration, and bone consolidation.

All reconstructions were performed during a single operative session by a combined orthopedic and plastic surgery team experienced in microsurgical reconstruction. At the beginning of the procedure, the orthopedic surgery team performed extensive debridement of infected bone and soft tissues, with revision, removal, or replacement of fixation devices when required. Concurrently, the plastic surgery team harvested a free VFG from the contralateral leg, with an associated fasciocutaneous flap when simultaneous bone and soft-tissue reconstruction was required (Figs. 1, 2, and 3).

1. *Donor site preparation.* A segment of the fibula was harvested from the contralateral (or sometimes ipsilateral) limb while preserving at least 5 cm of the proximal fibula and 7 cm of the distal fibula to maintain knee and ankle stability. The VFG was dissected meticulously together with its vascular pedicle based on the fibular artery, the fibular periosteum and, when needed for associated soft-tissue defects, an adjacent flap including a skin paddle (Taylor et al., 1975; Weiland et al., 1983).
2. *Recipient site preparation and graft implantation.* Following radical debridement, the recipient site was prepared to receive the graft. The vascularized fibular graft was inserted into the proximal and distal healthy-bone segments to bridge the defect and was fixed using internal or external fixation to achieve adequate stability. Internal fixation usually consisted of interfragmentary screws and/or locking-plate fixation. External fixation, when performed, used circular or monolateral constructs according to defect characteristics and surgeon preference. Microsurgical end-to-end or end-to-side vascular anastomoses were performed between the graft pedicle and recipient vessels to ensure graft perfusion. Recipient vessels were selected according to local vascular anatomy, with preferential use of the posterior tibial vessels whenever feasible. Adequate graft perfusion was confirmed intraoperatively before wound closure.
3. *Postoperative management.* Direct monitoring of graft perfusion was possible when an osteocutaneous flap including a skin paddle had been harvested. Otherwise, routine postoperative care was performed. Progressive weight-bearing and rehabilitation were supervised by the orthopedic team and started when clinical and radiological evidence allowed it. Systemic antibiotic therapy was continued after surgery and adjusted according to microbiological findings from intraoperative samples under the supervision of infectious-disease specialists.

### 2.5 Data collection

Clinical data were retrospectively extracted from electronic medical records and recorded in a secure pseudonymized database. Collected variables included patient characteristics (age, sex, smoking status, alcohol use, and comorbidities such as diabetes, obesity, and cardiovascular disease), trauma characteristics (fracture type [open or closed], anatomical location, and time from trauma to VFG), surgical history (number of previous procedures, previous reconstructive techniques such as the Masquelet technique, and fixation method used during VFG), and infection characteristics (microbiological findings, polymicrobial versus monomicrobial infection, and antibiotic therapy at the time of VFG).

### 2.6 Outcome assessment

The primary endpoint was treatment success, defined as the combination of bone consolidation and infection remission. Bone consolidation was defined as radiographic bridging of at least three of the four cortices on orthogonal radiographs, associated with painless full weight-bearing. Direct consolidation was defined as union achieved after VFG without secondary bone grafting. Radiographic assessment was independently performed by two experienced orthopedic surgeons. In cases of disagreement, the final assessment was established by consensus. Computed tomography (CT) was performed when radiographic assessment remained uncertain.

Infection remission was defined as the absence of clinical signs of recurrent infection, including local inflammation, wound drainage, or sinus tract formation; no requirement for additional surgical procedures related to recurrent infection; and no requirement for ongoing suppressive antimicrobial therapy at the last available follow-up. Recurrent infection was defined according to the FRI Consensus Definition. Bone consolidation, infection remission, and overall treatment success were analyzed separately.

Secondary outcomes included amputation, death, persistent non-union, secondary bone grafting, and donor-site or graft-specific complications. A targeted review of the medical records was performed to retrieve donor-site and limb-related complications (leg length discrepancy, lower-limb malformation, and sensory deficit), as well as graft-specific and fixation-related complications (fixation plate fracture, graft nonunion, graft failure, and arteriovenous thrombosis).

For time-to-event analyses, follow-up duration was defined as the interval from vascularized fibular grafting to bone consolidation, a competing event (amputation or death), or the last available clinical follow-up for patients without consolidation.

### 2.7 Statistical analysis

Statistical analyses were performed using R software (version 4.3.1). Continuous variables were expressed as medians (interquartile range [IQR]), and categorical variables were expressed as counts and percentages. Comparisons between groups were performed using the Mann–Whitney U test for continuous variables and Fisher's exact test for categorical variables.

The primary outcome was time to bone consolidation. Time origin was defined as the date of vascularized fibular grafting (VFG). Follow-up duration was calculated from VFG to bone consolidation, amputation, death, or last available clinical follow-up, whichever occurred first.

Bone consolidation was considered to be the event of interest. Amputation and death were treated as competing events because they precluded subsequent consolidation. Patients without consolidation or a competing event were censored at their last available follow-up.

The cumulative incidence of bone consolidation was estimated using a competing-risk framework, with amputation and death being considered to be competing events. Cumulative incidence functions and corresponding 95 % confidence intervals were estimated using the cmprsk package.

Given the limited sample size and number of events, no regression analysis was performed to identify predictors of bone consolidation. Secondary bone grafting was considered to be a post-baseline therapeutic intervention and was therefore described descriptively. No formal comparative analysis was performed between patients who underwent VFG alone and those who subsequently required secondary bone grafting.

All statistical analyses were descriptive or exploratory. Where applicable, two-sided p values < 0.05 were considered to be statistically significant.

## 3 Results

### 3.1 Study population

**Figure 4:**
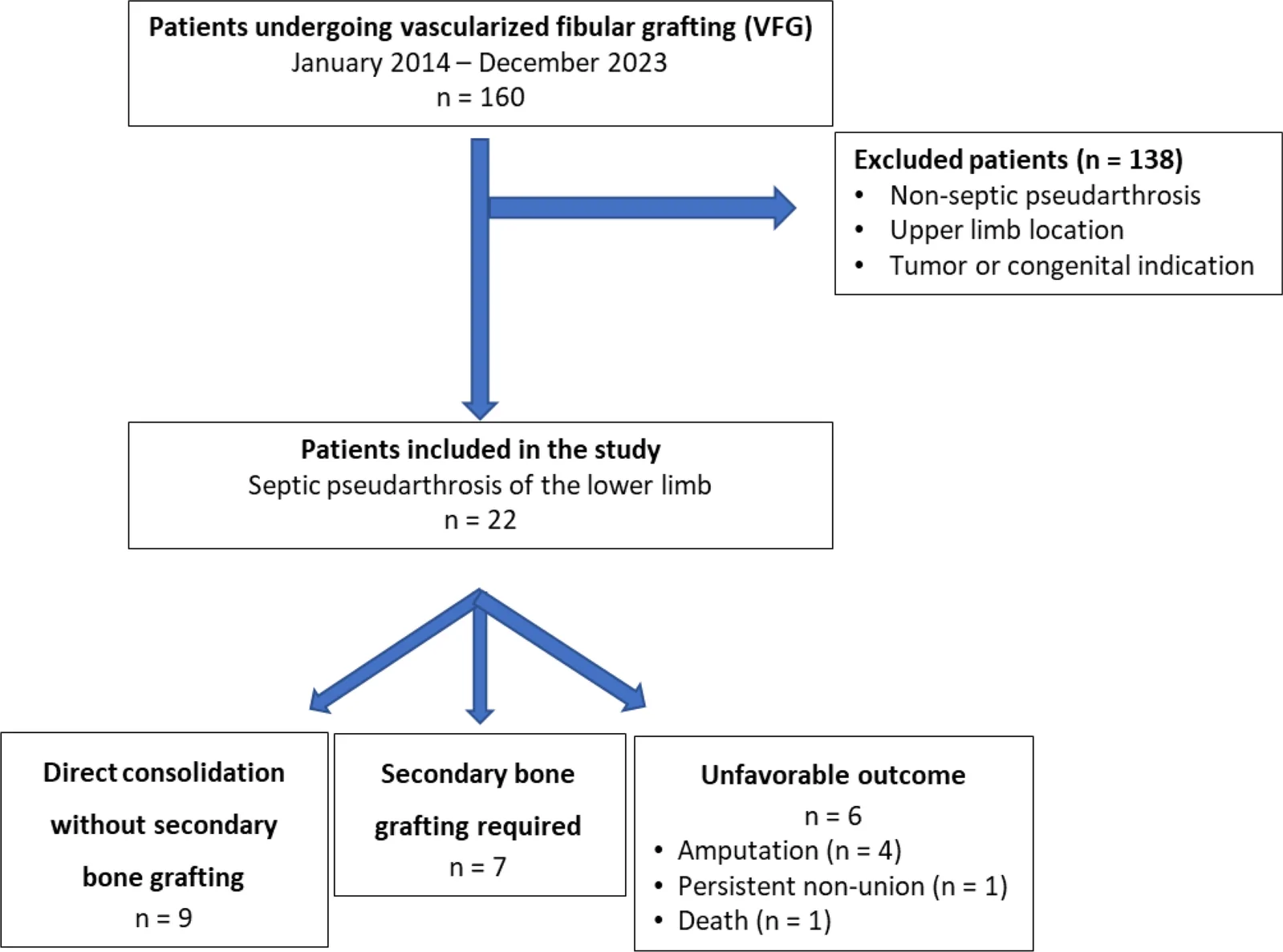
Study flowchart showing patient selection. A total of 22 patients with septic pseudarthrosis of the lower limb were included in the final analysis.

Between January 2014 and December 2023, 160 patients underwent vascularized fibular grafting (VFG) at our center. After applying inclusion and exclusion criteria, 22 patients with infected non-union of the lower limb were included (Fig. 4).

A total of 21 patients (95 %) had post-traumatic infected non-union, whereas one patient developed chronic infection following previous oncologic reconstruction for a femoral metastasis. This case was retained because the indication for VFG was management of an infected non-union after infection control surgery rather than primary tumor reconstruction.

The cohort comprised 16 men (73 %) and 6 women (27 %), with a median age of 45 years (IQR 36–52; range 18–74). Most injuries resulted from high-energy trauma, mainly road traffic accidents (86 %). Open fractures were present in 18 patients (82 %). The median interval between the initial trauma and VFG was 14 months (IQR 6–37 months), and patients had undergone a median of four previous surgical procedures.

The wide range in the interval between trauma and VFG was driven by one patient who underwent reconstruction more than 15 years after the initial open fracture (Table 1).

**Table 1:** Baseline characteristics according to treatment success.

Characteristic	Overall	Treatment success	No treatment	p value
	( n=22 )	( n=16 )	success ( n=6 )	
Sex				0.63
Female	6 (27 %)	5 (31 %)	1 (17 %)	
Male	16 (73 %)	11 (69 %)	5 (83 %)	
Age (Median, IQR)	45 years (IQR 36–52)	42.5 years (IQR 36–52)	49.5 years (IQR 45–58)	0.34
Any comorbidity				
Smoking	9 (41 %)	7 (44 %)	2 (33 %)	1.00
Diabetes	2 (9 %)	1 (6 %)	1 (17 %)	0.48
Obesity	4 (18 %)	3 (19 %)	1 (17 %)	1.00
Arterial hypertension	3 (14 %)	2 (12 %)	1 (17 %)	1.00
Peripheral arterial disease	2 (9 %)	0 (0 %)	2 (33 %)	0.065
Initial lesion				
Open fracture	18 (82 %)	14 (88 %)	4 (67 %)	0.29
Closed fracture	3 (14 %)	2 (12 %)	1 (17 %)	1.00
Non-traumatic (tumor/metastasis)	1 (5 %)	0 (0 %)	1 (17 %)	0.27
Mechanism of injury				
Motorcycle accident	13 (59 %)	9 (56 %)	4 (67 %)	1.00
Other road traffic accident	6 (27 %)	5 (31 %)	1 (17 %)	0.63
Ballistic injury	1 (5 %)	1 (6 %)	0 (0 %)	1.00
Horse-riding accident	1 (5 %)	1 (6 %)	0 (0 %)	1.00
Tumor-related reconstruction	1 (5 %)	0 (0 %)	1 (17 %)	0.27
Localization				0.65
Femur	9 (41 %)	6 (38 %)	3 (50 %)	
Tibia	13 (59 %)	10 (62 %)	3 (50 %)	
Microbiology				0.54
Monomicrobial infection	4 (18 %)	4 (25 %)	0 (0 %)	
Polymicrobial infection	18 (82 %)	12 (75 %)	6 (100 %)	
Previous surgical procedures before VFG	4 (2–5)	4 (2–4)	3.5 (3–5)	0.85
Time from infection diagnosis to VFG (Median, IQR)	22 d (3–108)	33 d (3–135)	7 d (4–12)	0.22
Time from trauma to VFG (Median, IQR)	14 months (10–36)	16 months (11–41)	12 months (11–14)	0.45

Values are median (IQR) or n (%). Baseline characteristics were compared between patients with and without overall treatment success using Fisher's exact test for categorical variables and the Mann–Whitney U test for continuous variables. All counts and p values were recalculated from patient-level source data.

### 3.2 Surgical history and previous reconstruction attempts

Before VFG, all patients had undergone multiple surgical interventions, including repeated debridement, fixation revisions, and reconstructive attempts. Nine patients (41 %) had previously received Masquelet induced-membrane reconstruction, which ultimately failed. These patients represented a particularly complex subgroup characterized by multiple prior surgical procedures and persistent infected bone defects, further supporting the use of VFG as a salvage reconstruction strategy.

### 3.3 Characteristics of bone defects and VFG reconstruction

The median bone defect size was 90 mm (range, 40–150 mm), and the median vascularized fibular graft length was 115 mm (range, 70–200 mm). Fibular grafts were harvested from the contralateral limb in 19 patients (86 %), from the ipsilateral limb in 2 patients (9 %), and bilaterally in 1 patient (5 %). Bone-only vascularized fibular grafts were used in 16 patients (73 %), whereas composite grafts were used in 6 patients (27 %), including osteocutaneous grafts in 2 patients (9 %), an osteomuscular graft in 1 patient (5 %), and other composite reconstructions in 3 patients (14 %). Detailed surgical characteristics of vascularized fibular grafting are summarized in Table 2.

**Table 2:** Surgical characteristics of vascularized fibular grafting ( n=22 ).

Characteristic	Overall ( n=22 )
Bone defect size, median (IQR)	90 mm (60–120)
Fibular graft length, median (IQR)	115 mm (80–150)
Origin of vascularized fibular graft	
Contralateral fibula	19 (86 %)
Ipsilateral fibula	2 (9 %)
Bilateral harvest	1 (5 %)
Type of vascularized fibular graft	
Bone-only graft	16 (73 %)
Osteocutaneous graft	2 (9 %)
Osteomuscular graft	1 (5 %)
Other composite reconstruction*	3 (14 %)
Definitive fixation	
External fixation	15 (68 %)
Internal fixation	7 (32 %)
Plate fixation	5 (23 %)
Intramedullary nail	1 (5 %)
Screw fixation	1 (5 %)
Antibiotic status at VFG	
Receiving antibiotic therapy	16 (73 %)
Antibiotic-free interval before VFG	6 (27 %)

Values are presented as median (IQR) or n (%). ^*^ Includes composite vascularized fibular graft reconstructions not classified as osteocutaneous or osteomuscular grafts, including one reconstruction combined with a structural femoral allograft.

Definitive stabilization was achieved using external fixation in 15 patients (68 %) and internal fixation in 7 patients (32 %), including plate fixation in 5 patients, intramedullary nailing in 1 patient, and screw fixation in 1 patient. At the time of reconstruction, 16 patients (73 %) were receiving antibiotic therapy, whereas 6 patients (27 %) underwent an antibiotic-free interval before VFG.

### 3.4 Microbiological findings

Overall, 18 patients (82 %) had a history of polymicrobial infection, and 4 (18 %) had monomicrobial infection based on microbiological documentation obtained during the course of infection management.

The most frequently isolated pathogens included *Staphylococcus aureus*, coagulase-negative staphylococci, and Gram-negative bacilli such as *Pseudomonas aeruginosa* and Enterobacterales. Anaerobic organisms, including *Clostridium* spp. and *Cutibacterium* *acnes*, were identified in several cases. One infection involved *Candida albicans*.

Antimicrobial resistance was uncommon and included methicillin-resistant *S. aureus* (MRSA) and resistance to fluoroquinolones or trimethoprim-sulfamethoxazole among Enterobacterales.

### 3.5 Antibiotic therapy

Antimicrobial therapy was supervised by infectious-disease specialists and adapted according to intraoperative microbiological findings. Treatment duration was individualized according to surgical management, microbiological results, and clinical evolution.

The median duration of postoperative antimicrobial therapy was 22 weeks (IQR 7–33 weeks).

Information regarding long-term suppressive antimicrobial therapy at final follow-up was reviewed. No patient was receiving suppressive antimicrobial therapy at the last follow-up visit.

### 3.6 Clinical outcomes

Direct consolidation without secondary grafting was observed in nine cases (41 %), with a median time to consolidation of 14 months (IQR 7–18). Consolidation following secondary bone grafting occurred in seven patients (32 %), with a longer median time to consolidation of 38 months (IQR 28–49 months). One additional patient achieved radiographic consolidation but experienced persistent infection and subsequently underwent amputation at the patient's request. Overall, bone consolidation was achieved in 17 patients (77 %).

At the last available follow-up, infection remission was present in 16 patients (73 %), and there was overall treatment success, defined as the combination of bone consolidation and infection remission, in 16 patients (73 %). Four patients underwent amputation during follow-up. One patient had persistent infection for more than 3 years despite early radiographic progression toward consolidation and requested amputation. A second patient underwent amputation because of persistent non-union associated with non-remission of infection. A third patient underwent amputation because of persistent infection despite early radiographic progression toward consolidation, associated with persistent pain and major depression. The fourth patient underwent amputation because of arterial and venous thrombosis leading to flap compromise and secondary necrosis.

A targeted review of the medical records identified additional donor-site, limb-related, and graft-specific complications. Donor-site and limb-related complications included leg length discrepancy in five patients, with a maximum discrepancy of 65 mm; lower-limb malformation in two patients; and sensory deficit in one patient. Graft-specific and fixation-related complications included fixation-plate fracture requiring surgical revision in one patient, graft non-union requiring multiple reoperations in one patient, graft failure in one patient, and arteriovenous thrombosis leading to flap compromise and subsequent amputation in two patients. 

### 3.7 Secondary bone grafting

Secondary bone grafting was performed in seven patients because of delayed consolidation despite maintained graft viability and infection remission. The median interval between VFG and secondary bone grafting was 12 months (IQR 11–16 months; range 5–16 months). All seven patients subsequently achieved bone consolidation. Given the small number of patients and the post-baseline nature of secondary bone grafting, no formal regression analysis was performed, and this observation should not be interpreted as evidence of a causal benefit of secondary bone grafting.

### 3.8 Time-to-event analysis and competing-risk analysis

**Figure 5:**
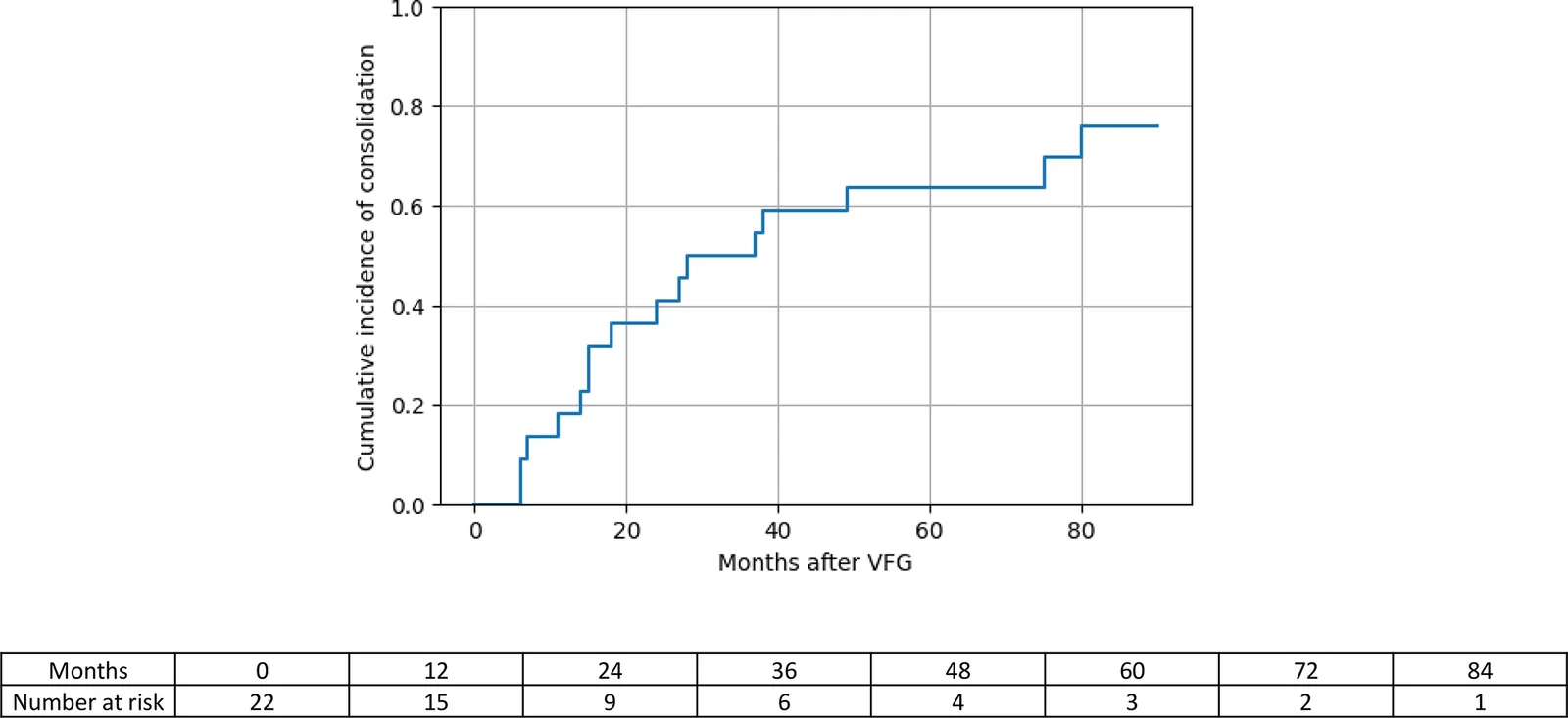
Overall cumulative incidence of bone consolidation after vascularized fibular grafting, accounting for amputation and death as competing events. Shaded areas represent 95 % confidence intervals. Numbers at risk are shown below the graph.

Cumulative incidence analysis, accounting for amputation and death as competing events, demonstrated a progressive increase in the probability of bone consolidation over time (Fig. 5).

The cumulative incidence of consolidation reached approximately 18 % at 12 months, 41 % at 24 months, 59 % at 36 months, and approximately 76 % at the end of follow-up. Most consolidation events occurred during the first 3 years after VFG, although late consolidation events were also observed. Five competing events occurred during follow-up, including four amputations and one death. These findings indicate that bone healing after VFG may require prolonged follow-up and that early assessment may underestimate the cumulative probability of consolidation.

## 4 Discussion

In the present study, vascularized fibular grafting (VFG) used as a salvage procedure for infected non-union of the lower limb resulted in a progressive increase in the cumulative incidence of consolidation over time, reaching 18 % at 12 months, 41 % at 24 months, 59 % at 36 months, and 76 % by the end of follow-up. Although lower than rates reported in non-infected settings, these findings reflect the extreme complexity of the population, characterized by chronic infection, multiple prior surgical failures, and compromised local biological conditions.

Infected non-union remains one of the most challenging complications in fracture-related infections. Chronic infection induces progressive bone destruction, impaired vascularity, and soft-tissue damage, all of which significantly compromise bone-healing potential. Management therefore requires a comprehensive strategy combining radical debridement; stable fixation; soft-tissue reconstruction; and prolonged, targeted antimicrobial therapy (Metsemakers et al., 2018).

Well-established host- and injury-related risk factors such as smoking, diabetes, obesity, and open fractures further contribute to poor outcomes by impairing both osteogenesis and immune response (Cicero-Álvarez et al., 2016; Jensen et al., 2022). These factors were prevalent in our cohort, reflecting the severity and complexity of cases referred to a tertiary center for limb-salvage reconstruction.

Several reconstructive techniques are available for large-bone defects, including the Masquelet induced-membrane technique and callus distraction with bone transport (Giovanoulis et al., 2023; Metsemakers et al., 2026; Sidiropoulos et al., 2023). Outcomes following distraction osteogenesis vary according to defect size, soft-tissue conditions, fixation strategy, and patient-related factors. These techniques have demonstrated satisfactory results in appropriately selected patients and remain important reconstructive options for fracture-related infection. However, they rely primarily on the regenerative capacity of the recipient site and may be more challenging in the presence of extensive scarring, compromised vascularity, or repeated surgical failures. Notably, a substantial proportion of our patients had previously undergone unsuccessful reconstructive attempts, including Masquelet procedures, underscoring the role of VFG as a salvage strategy in highly selected and complex cases.

The biological advantage of VFG lies in the transfer of living bone tissue with its intrinsic vascular supply, as initially described by Taylor et al. (1975). This vascularization enables graft viability even in hostile environments and promotes osteogenesis, remodeling, and resistance to infection (Malizos et al., 1993; Weiland et al., 1983). In addition, preserved microcirculation may enhance local immune response and antibiotic delivery, which is particularly relevant in chronic osteomyelitis and biofilm-associated infections (Trampuz and Widmer, 2006; Zimmerli and Sendi, 2011).

Another important technical consideration is the length of the fibular graft, which allows reconstruction of large segmental defects that may exceed the limits of other techniques. In selected cases, a staged or hybrid reconstructive strategy can be considered, combining VFG with secondary procedures such as additional cancellous bone grafting or partial Masquelet-type reconstruction to enhance consolidation at specific interfaces. This flexibility further supports the role of VFG as a cornerstone technique in complex limb-salvage situations.

In the literature, consolidation rates following VFG range from 70 % to 90 % in heterogeneous cohorts including trauma, tumor, and congenital indications (Feltri et al., 2023; Minami et al., 2000) and approximately 60 % to 80 % in more recent series (Smolle et al., 2024). However, these studies often include non-infected cases and therefore are not directly comparable to the present cohort. By contrast, our study focused exclusively on infected non-union after multiple surgical failures, which likely explains the lower early success rate observed.

Importantly, our findings highlight that VFG outcomes are strongly time-dependent. At 12 months, the cumulative incidence of consolidation was approximately 18 %, increasing progressively to 76 % by the end of follow-up. This delayed but continuous improvement highlights the prolonged nature of healing after VFG. From a clinical perspective, these findings support maintaining a reconstructive strategy over time, particularly when functional improvement is achieved, before considering treatment failure or limb amputation.

Secondary bone grafting was performed in seven patients because of delayed consolidation, and all subsequently achieved bone consolidation. However, secondary bone grafting was a post-baseline intervention performed selectively according to the clinical evolution of individual patients. Therefore, this observation should not be interpreted as evidence of a causal benefit. Rather, it illustrates that a staged reconstructive approach, including additional bone grafting when clinically indicated, may be feasible in selected patients with persistent delayed consolidation.

A major strength of this study is the use of a competing-risk framework. In this clinical setting, amputation and death preclude the occurrence of bone consolidation and should therefore not be treated as censored observations. Traditional Kaplan–Meier methods may overestimate consolidation rates by ignoring these competing events. By contrast, cumulative incidence functions provide a clinically relevant estimation of consolidation by accounting for competing events.

Despite its biological advantages, VFG remains a technically demanding procedure requiring microsurgical expertise and careful patient selection. Complications such as vascular thrombosis, graft fracture, delayed union, and donor-site morbidity must be considered. Moreover, patients with severe peripheral vascular disease or poor overall condition may not be suitable candidates.

Our study also reinforces the importance of multidisciplinary management in complex bone infections. Close collaboration between orthopedic surgeons, plastic surgeons, infectious-disease specialists, and microbiologists is essential to optimize surgical strategy, antimicrobial therapy, and follow-up. Such coordinated care is a key component of successful limb salvage in these patients.

Another consideration when interpreting our findings is the heterogeneity of indications. Although the cohort mainly consisted of post-traumatic infected non-unions, one patient presented with chronic infection following previous oncologic reconstruction. This case was retained because the indication for VFG was management of an infected non-union after infection eradication rather than primary tumor reconstruction itself. Given that only one such case was included, its impact on the overall results is likely to be negligible.

This study has several limitations. First, its retrospective design and small sample size limit statistical power and preclude reliable identification of predictors of bone consolidation. Second, the retrospective nature of the study resulted in incomplete and heterogeneous documentation of several clinically relevant variables. Donor-site and graft-specific complications were not systematically recorded in the medical charts and may therefore have been underestimated. Similarly, detailed information regarding the source and type of secondary bone grafts, grafting techniques, fixation revisions, microbiological findings at the time of secondary grafting, and perioperative antimicrobial management was not consistently available and could not be analyzed comprehensively. Information regarding the route and sequence of antimicrobial administration, including intravenous and oral therapy, was also not systematically available.

Third, follow-up duration was heterogeneous across the cohort. Observation time was defined as the interval from vascularized fibular grafting to consolidation, amputation, death, or last available follow-up. The median observation time was 21.5 months (IQR 15–49 months; range 1–90 months), while several patients were followed for more than five years and one patient for up to 90 months. This variability justified the use of competing-risk and time-to-event analyses and allowed the capture of late consolidation events, particularly following secondary bone grafting. Nevertheless, the relatively limited duration of follow-up in some patients may have influenced long-term outcome estimates.

Although restoration of functional mobility is an important goal of limb-salvage procedures, functional outcomes were not systematically collected in this retrospective cohort. Therefore, no conclusions can be drawn regarding postoperative functional recovery, quality of life, or patient satisfaction. Future prospective studies should evaluate these outcomes alongside radiological and infection-related endpoints.

Despite these limitations, this study provides clinically relevant data on the use of VFG as a salvage strategy in one of the most challenging orthopedic settings. By specifically focusing on infected non-union and applying a competing-risk framework, our findings contribute to a more accurate understanding of the timing and probability of bone consolidation. Larger multi-center studies with standardized functional outcome assessment are needed to confirm these findings and further define the role of VFG and adjunctive reconstructive procedures in the management of complex infected non-union.

## 5 Conclusions

Vascularized fibular grafting appears to be a valuable limb-salvage option for selected patients with complex infected non-unions of the lower limb. In this retrospective single-center study, consolidation occurred progressively over time and often required prolonged follow-up. Given the limited sample size and exploratory nature of the analyses, larger prospective multi-center studies are needed to confirm these findings and better define the role of adjunctive procedures such as secondary bone grafting.

## Data Availability

The underlying research data are not publicly accessible. The study is based on retrospective clinical data from patients treated at Assistance Publique – Hôpitaux de Marseille (AP-HM). Individual-level clinical data cannot be made publicly available because of patient confidentiality, privacy requirements, and the applicable institutional and regulatory framework governing access to clinical research data, in accordance with the ethics approval obtained for this study (see Ethics Approval section). No third-party dataset was used; all data originated from a single institutional source. The anonymized study data are securely stored within the institutional framework and may be made available upon reasonable request to the corresponding author, subject to institutional approval and applicable data protection regulations.

## Code availability

The statistical analyses were performed using R software (version 4.3.1), including the open-source cmprsk package for competing-risk analysis. The underlying analysis code consists of a custom R script using standard functions from R and the cmprsk packages; no proprietary software or proprietary algorithms were used.

The analysis code is not currently deposited in a public repository as no dedicated repository was established at the time of submission. It is, however, available from the corresponding author upon reasonable request. The R software and cmprsk package used for the analyses, including their version numbers, are described in the Methods section and cited in the reference list. R software is freely available from the R Project website (https://www.r-project.org/, last access: 14 September 2026), and the cmprsk package is freely available from CRAN (https://CRAN.R-project.org/package=cmprsk, last access: 22 August 2026).
